# Supplementary material for: Severity, mortality, long COVID-19, and quality of life: Insights from a cohort study of hospitalized COVID-19 patients during the delta variant predominance period in Thailand
Source: PLoS One. 2025 Jun 13;20(6):e0324061. doi: 10.1371/journal.pone.0324061 (PMC12165381; doi:10.1371/journal.pone.0324061)
Supplement: Attachment — Submitted filename: Response to Reviewers.docx [file pone.0324061.s003.docx]

**Response to Reviewers**

**December 10, 2024**

Author: Pimpinan Khammawan

**Title:** Severity, Mortality, Long COVID-19, and Quality of Life: Insights from a Cohort Study of Hospitalized COVID-19 Patients in a Thai Tertiary Care Hospital

**Revise Title:** Severity, Mortality, Long COVID-19, and Quality of Life: Insights from a Cohort Study of Hospitalized COVID-19 Patients during the Delta Variant Predominance period in Thailand

| Reviewer Comment | Revisions | page |
| --- | --- | --- |
| Reviewer #1:   1. There are grammatical mistakes in the manuscript. I recommend the authors to undertake a thorough review of the manuscript taking the help of someone fluent in the English language to correct them. This will make the submission more articulate. | Reviewer #1   1. Grammatical Errors: Thank you for pointing out the grammatical mistakes. I will thoroughly review the manuscript and seek assistance from a fluent English speaker or a professional editing service to ensure that the language is clear, correct, and unambiguous. |  |
| Reviewer #2:   1. Abstract: The abstract should be revised to include information on mortality and the clinical characteristics of Long COVID. Additionally, the abbreviations for quality of life (PCS, MCS) should be clarified. 2. Introduction: The authors should address the research gap and clearly state the objective of the study. 3. Method: What is the study population? The authors state, “the study population consists of moderate to severe COVID-19 patients discharged from Nakornping Hospital between July and December 2021.” Please ensure the data is consistent with the results, particularly regarding patients who died during admission. All research tools should be described in greater detail, including their interpretation and validation. Who conducted the interviews, and how many interviewers were involved? The statistical analysis section should clearly outline the methods used for comparing QoL scores. The logistic regression exploring associations with Long COVID needs verification, as the authors' description lacks clarity and does not include the results. Please confirm whether multivariate analysis or multivariable analysis was used for this study. 4. Results: The results section requires significant revision. The authors should clarify the number or sample size used for the analysis. What severity levels are presented: moderate vs. severe? Were patients who died during admission included in the analysis? Which death cases were used in Table 3—those who died during admission or after discharge? The odds ratios (OR) for each factor and the reporting of p-values should be checked for accuracy, particularly for variables such as age and vaccination. Table 4 should also be rechecked, as some variables report p-values inappropriately, and the scales or characteristics of the data that are appropriate for statistical analysis, such as length of stay, should be reconsidered. 5. Discussion: The discussion cannot provide meaningful comments at this stage because the study results need to be thoroughly checked. | Reviewer #2   1. Abstract:  - The abstract will be revised to include specific information on mortality rates and the clinical characteristics of Long COVID. - Abbreviations such as PCS and MCS will be clarified to ensure readers understand their significance.  2. Introduction:  - address the research gap and explicitly state the objective of the study to make the purpose of our research more evident.  3. Methodology:    - ensure the study population is clearly defined and consistent with the results section, particularly with respect to patients who died during admission.    - Research tools will be described in greater detail, including their interpretation and validation processes.    - Details about the interview process, including the number and training of interviewers, will be added.    - The statistical analysis section will be revised to provide clear details about the methods used to compare QoL scores. I will confirm whether a multivariate or multivariable analysis was used and ensure this is clearly stated.    - The logistic regression section will be reviewed to enhance clarity and include all relevant results. 4. Results:  - The sample size and severity levels (moderate vs. severe) will be clarified. - verify whether patients who died during admission were included and specify how death cases are represented in Table 3. - Odds ratios (OR) and p-values for variables such as age and vaccination will be checked for accuracy. - Table 4 will be reviewed to ensure appropriate reporting of p-values and data scales.  5. Discussion:  - The discussion will be revised after verifying and refining the study results to ensure that the commentary aligns with accurate data. | Page2  Background  Finding  Introduction  Page 3,4  Methodology  Page5,6  Results  Page 13  Table4  Page 7  discussion |
| Reviewer #3:  Review of the article: Severity, Mortality, Long COVID-19, and Quality of Life: Insights from a Cohort Study of Hospitalized COVID-19 Patients in a Thai Tertiary Care Hospital Thank you very much for allowing us to review this interesting article. The study was reviewed according to the STROBE guidelines. It is very well written and organized.  It is important to mention that the calculation of sample size should be included in the study, as analytical calculations were performed regarding the factors that impacted severity, mortality, and quality of life.  The main objective is not highlighted in the abstract nor in the main document.  Additionally, since baseline quality of life was not evaluated, and no scale was applied to assess resilience, it cannot be asserted with certainty that the patients were resilient.  I would like to ask whether the included patients were consecutive admissions of COVID patients, and the indications for invasive mechanical ventilation, as it is striking that the mortality of ventilated patients reported in the study (11%) is considerably lower than what is reported in the global literature, where most series reported more than 50% and even up to 80% in this type of patient, considering that the main indication for mechanical ventilation was ARDS (acute respiratory distress syndrome). | Reviewer #3   1. Sample Size Calculation:    - A detailed explanation of the sample size calculation will be included to align with the analytical objectives of the study. 2. Objective of the Study:    - The main objective will be clearly highlighted in both the abstract and the main document. 3. Baseline Quality of Life and Resilience:    - I acknowledge the limitation regarding baseline QoL and resilience. This will be mentioned in the limitations section, and we will clarify that the assertion of resilience is tentative. 4. Patient Admissions and Mechanical Ventilation:   **This study had 2 classify of the covid-19 patient who need to use the ventilator**  **Define: Critical COVID-19:** This designation is characterized by the presence of criteria indicative of acute respiratory distress syndrome (ARDS), sepsis, septic shock, or other conditions necessitating life-sustaining interventions such as mechanical ventilation (either invasive or non-invasive) or vasopressor therapy.  **Define: Severe COVID-19:** This classification encompasses any of the following criteria:   - Oxygen saturation < 90% on room air. - In adults, it includes indications of severe respiratory distress (e.g., utilization of accessory muscles, inability to articulate complete sentences, respiratory rate > 30 breaths per minute), while in children, it involves severe chest wall in-drawing, grunting, central cyanosis, or the presence of general danger signs (e.g., inability to breastfeed or ingest liquids, lethargy or reduced level of consciousness, convulsions) in addition to signs of pneumonia.   The result shown, severe case 87 cases, death during admit 39 cases, death after discharge 20 cases and alive just 28 cases (table 1) | Page3,4  Study design  Page2  Background  Page8  Strengths and limitations  Page11  Table1 |
