# Supplementary material for: Severity, mortality, long COVID-19, and quality of life: Insights from a cohort study of hospitalized COVID-19 patients during the delta variant predominance period in Thailand
Source: PLoS One. 2025 Jun 13;20(6):e0324061. doi: 10.1371/journal.pone.0324061 (PMC12165381; doi:10.1371/journal.pone.0324061)
Supplement: Attachment — Submitted filename: Response_to_Reviewers_auresp_2.docx [file pone.0324061.s004.docx]

**Response to Reviewers**

**February 25, 2025**

Author: Pimpinan Khammawan

**Title:** Severity, Mortality, Long COVID-19, and Quality of Life: Insights from a Cohort Study of Hospitalized COVID-19 Patients during the Delta Variant Predominance period in Thailand

**Revise Title:** -

| Reviewer Comment | Revisions | page |
| --- | --- | --- |
| **3. Has the statistical analysis been performed appropriately and rigorously?** |  |  |
| Reviewer #1:   1. Yes | Reviewer #1:   1. - |  |
| Reviewer #2: - | Reviewer #2: - |  |
| Reviewer #3:   1. No | Reviewer #3:  Data were analyzed using SPSS version 26.0 (SPSS Inc., Chicago, IL, USA). Descriptive statistics summarized general characteristics using sums, percentages, means, medians (IQR), and Analytical statistics use Chi-square test (χ² test) or Fisher’s Exact test is used to compare the proportions of categorical variables, such as gender, age, vaccination status, occupation, ventilator use, and length of hospital stay, with survival status. This test determines whether these variables are associated with mortality rates and survival rates. Long-term physical and psychological symptoms and Quality of Life (QoL) were monitored for one- year post-hospitalization. Multivariate Logistic Regression use to analyzes multiple variables simultaneously while adjusting for confounding factors. Reports Adjusted Odds Ratio (AOR), 95% Confidence Interval (CI), and p-value and analyzed factors associated with Long COVID, including chronic conditions, with quantitative and qualitative variables examined through means, frequencies, percentages, and hypothesis testing and used to analyze the relationship between independent variables (Gender, Age, BMI, Severity Level, Comorbidity, Occupation) and the outcomes PCS (Physical Component Summary) and MCS (Mental Component Summary). Independent t-test used to compare the mean values of PCS and MCS between two groups of variables, such as: Gender (Male and Female), Disease severity (Moderate and Severe), Comorbidity (Yes and No), Length of hospital stay (≤ 2 weeks and > 2 weeks). | Page 5  Statistic |
| **6. Review Comments to the Author** |  |  |
| Reviewer #1: (No Response) | Reviewer #1: - |  |
| Reviewer #2: - | Reviewer #2: - |  |
| Reviewer #3:  **Abstract** There is a significant grammatical and writing error in the "Methods" section.  The study characterized 604 moderate to severe COVID-19 patients treated at a Tertiary Care Hospital during the Delta wave in Thailand (July–December 2021) by analyzing secondary data from medical records. Confirmed cases were monitored as a cohort using a Long COVID questionnaire to track symptoms, chronic conditions, and assess social impact a year after discharge. Quality of life, including physical and mental health, was evaluated using the SF-12 questionnaire.  The first time an acronym is used, its full meaning must be provided. | Reviewer #3:  **Abstract**  **(Methods)**  The study characterized 604 are moderate to severe COVID-19 patients at Tertiary Care Hospital during the Delta wave in Thailand (July-December 2021), using secondary data from medical records. Confirmed cases were cohort monitored using a Long COVID questionnaire for symptoms, chronic conditions, and social impact a year after discharge. Quality of life was evaluated using the SF-12 questionnaire (SF-12: 12-Item Short Form Survey). | Page 2  Abstract  (Methods) |
| **Introduction** The introduction must conclude with the clearly stated objective of the research. | This study have 3 major objective:  This study aimed to investigate the factors associated with Long COVID among hospitalized patients during the predominance of the Delta variant, to describe the characteristics of hospitalized COVID-19 patients during the Delta variant predominance and to assess the quality of life of patients after discharge from a tertiary care hospital in Chiang Mai Province. | Page 3  Introduction  (paragraph 5^th^) |
| **Statistical Analysis** Descriptive statistics are well presented, but it is unclear why and in which part of the results the chi-square or Fisher's exact test was used for comparing qualitative variables and among which groups. Analytical results are presented, not just descriptive ones, but there is no mention of the sample size calculation apart from its inclusion in the selection flowchart. | **Sample Size:**  The selection of cases for this study was purposive, selected all cases concentrating particularly on patients admitted to Nakornping Hospital during the period from July to December 2021. This sample size was 604 cases, as shown in figure 1.  The inclusion criteria for this study are as follows: 1. Confirmed positive for SAR-CoV-2 infection through RT-PCR testing, 2. Participants who were willing to participate, 3. Participants reachable by telephone for post-discharge follow-up evaluations and 4. Participants who were able to communication in the Thai language. | Page 4  Sample Size |
|  | **Statistics:**  Data were analyzed using SPSS version 26.0 (SPSS Inc., Chicago, IL, USA). Descriptive statistics summarized general characteristics using sums, percentages, means, medians (IQR), and Analytical statistics use Chi-square test (χ² test) or Fisher’s Exact test is used to compare the proportions of categorical variables, such as gender, age, vaccination status, occupation, ventilator use, and length of hospital stay, with survival status. This test determines whether these variables are associated with mortality rates and survival rates. Long-term physical and psychological symptoms and Quality of Life (QoL) were monitored for one- year post-hospitalization. Multivariate Logistic Regression use to analyzes multiple variables simultaneously while adjusting for confounding factors. Reports Adjusted Odds Ratio (AOR), 95% Confidence Interval (CI), and p-value and analyzed factors associated with Long COVID, including chronic conditions, with quantitative and qualitative variables examined through means, frequencies, percentages, and hypothesis testing and used to analyze the relationship between independent variables (Gender, Age, BMI, Severity Level, Comorbidity, Occupation) and the outcomes PCS (Physical Component Summary) and MCS (Mental Component Summary). Independent t-test used to compare the mean values of PCS and MCS between two groups of variables, such as: Gender (Male and Female), Disease severity (Moderate and Severe), Comorbidity (Yes and No), Length of hospital stay (≤ 2 weeks and > 2 weeks). | Page 5  Statistic |
| **Results** In Table 4, which shows the analysis of mental and physical components, it seems that a t-test was used to compare the mean score in each category. However, this is not reflected in the statistical analysis section. Additionally, there is no multivariate analysis to determine which independent factors were associated with worse quality-of-life scores. Was this not performed, or was it simply not shown? | Independent t-test used to compare the mean values of PCS and MCS  **multivariate analysis** to determine which independent factors were associated with worse quality-of-life scores shown in the Table 5: Factors Associated with Physical and Mental Component Summary (PCS and MCS) Scores in Hospitalized COVID-19 Patients | Page 5  Statistic  Page 7&13   - t-test - multivariate analysis |
