# Supplementary material for: Severity, mortality, long COVID-19, and quality of life: Insights from a cohort study of hospitalized COVID-19 patients during the delta variant predominance period in Thailand
Source: PLoS One. 2025 Jun 13;20(6):e0324061. doi: 10.1371/journal.pone.0324061 (PMC12165381; doi:10.1371/journal.pone.0324061)
Supplement: Attachment — Submitted filename: Response to Reviewers _4 April25.docx [file pone.0324061.s005.docx]

**Response to Reviewers**

**April 2, 2025**

Author: Pimpinan Khammawan

**Title:** Severity, Mortality, Long COVID-19, and Quality of Life: Insights from a Cohort Study of Hospitalized COVID-19 Patients during the Delta Variant Predominance period in Thailand

**Revise Title:** -

| **Reviewer Comment** | **Revisions** | **page** |
| --- | --- | --- |
| **Background (Abstract)** “The data collected from screening forms and patient history files were analyzed to highlight the clinical characteristics of Long COVID and its implications for patient well-being.” I believe this sentence is unnecessary in the background section of the abstract. The paragraph reads more clearly if it ends with the objectives. Moreover, this part belongs to the methods section. | **Remove:**  “Data collected from screening forms and patient history files were analyzed to highlight the clinical characteristics of Long COVID and its implications for patient well-being.”  **Rewrite:**  **Background:**  The COVID-19 pandemic, declared in March 2020, has had significant global impacts, with Thailand reporting over 4.6 million cases and 32,000 fatalities by September 2022. Long COVID, or Post-COVID Conditions (PCC), affects 10–30% of COVID-19 patients globally, with symptoms lasting beyond three months. Common issues include fatigue, brain fog, respiratory problems, and psychological effects such as anxiety and depression. Symptoms can persist regardless of the initial infection severity, and ongoing research continues to refine understanding and management strategies.  To address residual symptoms of COVID-19 during the Delta variant predominance period, a study was conducted from July to December 2021 at a tertiary care hospital in Chiang Mai, Thailand. The study aimed to describe the characteristics of COVID-19 patients, explore the Long COVID symptoms experienced by patients after discharge, and assess their quality of life. | **Page 2**  **Background (Abstract)** |
| **Findings (Abstract)** In the results reported in the abstract, the description begins with the population, followed by findings on factors associated with severity and mortality. However, the results regarding factors associated with Long COVID — which is the main objective — are not presented. This must be included, as it is the main focus and is not clear when reading the abstract. This final part of the findings is good and aligns well with the secondary objectives of the study: “One- year post-hospitalization, 79.15% had no Long COVID symptoms. Quality of life scores were high (Physical Component Summary: PCS = 48.62, Mental Component Summary: MCS = 50.65)” | **Findings:**  Most patients were Thai (85.9%) and female (57.3%), with obesity common among those aged 18-60 (48.3%). Severe cases and mortality were higher in patients over 60 (30.2%) and unvaccinated patients (60.4%). Severity was related with male gender, older age, lack of antiviral use, and being unvaccinated; overweight status, comorbidities, and abnormal chest x-rays were not significant. Deaths were influenced by gender, age, and antiviral use, but not hospital stay duration, overweight status, comorbidities, or vaccination status. At one-year follow-up, Long COVID symptoms were reported in a small proportion of patients (4.2% shortness of breath, 1.5% chronic cough), mostly in adults and older adults. Other symptoms were rare (<1%) and limited to the 18–60 age group. No severe neurological or systemic symptoms were reported. One-year post-hospitalization, 79.15% had no Long COVID symptoms. Quality of life scores were high (Physical Component Summary: PCS = 48.62, Mental Component Summary: MCS = 50.65).  . | **Page 2**  **Findings (Abstract)** |
| **Interpretation (Abstract)** In the interpretation section of the article, there should also be alignment with the main objective, which is to identify factors associated with Long COVID. This should be addressed in the conclusion. This final part of the interpretation is appropriate and relates to the study's secondary objectives: “Despite varied long COVID symptoms, most reported good physical and mental health one year after recovery. These findings highlight the resilience of patients and the importance of monitoring long-term health outcomes.” | **Interpretation:**  This study found a very low prevalence of Long COVID symptoms, which may be due to the severity of the Delta variant leading to higher mortality among patients with severe illness. Those who survived and recovered mostly had moderate symptoms and were predominantly under 60 years of age, which may explain the lower occurrence of Long COVID in this group.  The majority of COVID-19 patients in Chiang Mai experienced moderate symptoms and had a high survival rate. Despite varied long COVID symptoms, most reported good physical and mental health one year after recovery. These findings highlight the resilience of patients and the importance of monitoring long-term health outcomes. | **Page 3**  **Interpretation (Abstract)** |
| **Introduction** The introduction now more clearly states the objectives; however, it is advisable to present the objectives at the end of the introduction. The last paragraph of the introduction could be moved up before the objectives. | **Move the objective to the end of the introduction:**  Many COVID-19 patients require close monitoring, there are several gaps, such as limited understanding of disease mechanisms, variability in symptoms, and insufficient population-specific data. Long-term impacts on quality of life and the role of vaccines remain underexplored. Challenges to healthcare systems and factors influencing resilience and recovery also require further research to improve prevention, diagnosis, and disease management.  This study aimed to describe the characteristics of hospitalized COVID-19 patients during the Delta variant predominance and to assess the Long COVID and quality of life of patients after discharge from a tertiary care hospital in Chiang Mai Province. | **Page 3**  **Introduction** |
| **Methods** There should be a clear operational definition of Long COVID as a variable. | **Methods:**  The study characterized 604 are moderate to severe COVID-19 patients at Tertiary Care Hospital during the Delta wave in Thailand (July-December 2021), using secondary data from medical records. Confirmed cases were cohort monitored using a Long COVID questionnaire for symptoms, chronic conditions, and social impact a year after discharge. Quality of life was evaluated using the SF-12 questionnaire (SF-12: 12-Item Short Form Survey).  Long COVID, in this study, is defined as the persistence or emergence of one or more physical, psychological, or cognitive symptoms that last for more than 12 weeks after the initial onset of COVID-19 and cannot be explained by alternative diagnoses. This includes, but is not limited to, symptoms such as fatigue, dyspnea, chest pain, cough, cognitive dysfunction ("brain fog"), insomnia, anxiety, or depression. | **Page 2**  **Methods** |
| **Results** The results show a low rate of Long COVID, and the bulk of the analysis focuses on severity, mortality, and quality of life — that is, on secondary objectives. The main objective stated — “This study aimed to investigate the factors associated with Long COVID among hospitalized patients during the predominance of the Delta variant” — is not adequately addressed (possibly due to sample size limitations). | **Long COVID after discharge at one-year follow up** The table 4.6 presents symptoms experienced by COVID-19 patients in different age groups at one-year follow-up post-discharge among 331 patients. Shortness of breath is the most commonly reported symptom, affecting 14 patients (4.2%), with 1.1% in the <18 age group, 5.0% in the 18-60 age group, and 7.0% in the >60 age group. Chronic cough was reported by 5 patients (1.5%) overall, including 1.1% in the <18 group, 1.7% in the 18-60 group, and 1.8% in the >60 group. Other symptoms, such as palpitations, vertigo, loss of taste and smell, and hair loss, were each reported by only 1 patient (0.3%), all in the 18-60 age group. Additional symptoms, including poor sleep, brain fog, and constipation, were reported by 2 patients each (0.6%) in the 18-60 group. No cases were recorded for seizures, paraplegia, severe headache, change in voice, muscle or joint aches, rash, menstrual cycle changes, sore throat, chest congestion, chest pain, nausea/vomiting, or changes in the menstrual cycle across all age groups. Overall, the occurrence of Long COVID symptoms is relatively low, with the majority of symptoms affecting a small proportion of patients, primarily in the 18-60 and >60 age groups. **Add Table:** Table 4 Long COVID classify by age group after discharge at one-year follow up (n=331) **Variable** \| **Number (%)** \| \| \|; \| **Total** \| **<18 year** \| **18-60 year** \| **>60 year**; \| **(n=331)** \| **(n=94)** \| **(n=180)** \| **(n=57)**; Palpitation \| 1 (0.3) \| 0 (0.0) \| 1 (0.6) \| 0 (0.0); Seizures \| 0 (0.0) \| 0 (0.0) \| 0 (0.0) \| 0 (0.0); Poor sleep \| 2 (0.6) \| 0 (0.0) \| 2 (1.1) \| 0 (0.0); Brain fog \| 2 (0.6) \| 0 (0.0) \| 2 (1.1) \| 0 (0.0); Paraplegia \| 0 (0.0) \| 0 (0.0) \| 0 (0.0) \| 0 (0.0); Severe Headache \| 0 (0.0) \| 0 (0.0) \| 0 (0.0) \| 0 (0.0); Vertigo \| 1 (0.3) \| 0 (0.0) \| 1 (0.6) \| 0 (0.0); Loss of taste and smell \| 1 (0.3) \| 0 (0.0) \| 1 (0.6) \| 0 (0.0); Shortness of breath \| 14 (4.2) \| 1 (1.1) \| 9 (5.0) \| 4 (7.0); Chronic cough \| 5 (1.5) \| 1 (1.1) \| 3 (1.7) \| 1 (1.8); Change in voice \| 0 (0.0) \| 0 (0.0) \| 0 (0.0) \| 0 (0.0); Muscle or joint aches \| 0 (0.0) \| 0 (0.0) \| 0 (0.0) \| 0 (0.0); Rash \| 0 (0.0) \| 0 (0.0) \| 0 (0.0) \| 0 (0.0); Hair Loss \| 1 (0.3) \| 0 (0.0) \| 1 (0.0) \| 0 (0.0); Change in menstrual cycle \| 0 (0.0) \| 0 (0.0) \| 0 (0.0) \| 0 (0.0); Sore throat \| 0 (0.0) \| 0 (0.0) \| 0 (0.0) \| 0 (0.0); Chest congestion \| 0 (0.0) \| 0 (0.0) \| 0 (0.0) \| 0 (0.0); Chest pain \| 0 (0.0) \| 0 (0.0) \| 0 (0.0) \| 0 (0.0); Pain in abdomen \| 1 (0.3) \| 0 (0.0) \| 1 (0.6) \| 0 (0.0); Constipation \| 2 (0.6) \| 0 (0.0) \| 2 (1.1) \| 0 (0.0); Nausea/vomiting \| 0 (0.0) \| 0 (0.0) \| 0 (0.0) \| 0 (0.0); Diarrhea \| 1 (0.3) \| 0 (0.0) \| 1 (0.6) \| 0 (0.0) | **Page 7,14**  **Results**  **Table 4** |
| **In summary**, this is an interesting study that has improved compared to its previous version. It includes multivariate analyses; however, there is a lack of coherence between the stated main objective, the presentation of results, and the conclusions. It might be easier to restructure the order of the objectives than to rework the entire analysis to align it with the original main objective. | **Adjusted objective of study:**  **(Adjusted objective of study in Abstract (Background) and last paragraph in Introduction)**  This study aimed to describe the characteristics of hospitalized COVID-19 patients during the Delta variant predominance and to assess the Long COVID and quality of life of patients after discharge from a tertiary care hospital in Chiang Mai Province. | **Page 2,4**  **Abstract (Background),**  **Introduction** |
